# Supplementary material for: Predictability of Genetic Interactions from Functional Gene Modules
Source: G3 (Bethesda). 2016 Dec 21;7(2):617–24. doi: 10.1534/g3.116.035915 (PMC5295606; doi:10.1534/g3.116.035915)
Supplement: Supplementary file 1 [file 617FigureS1.pdf]

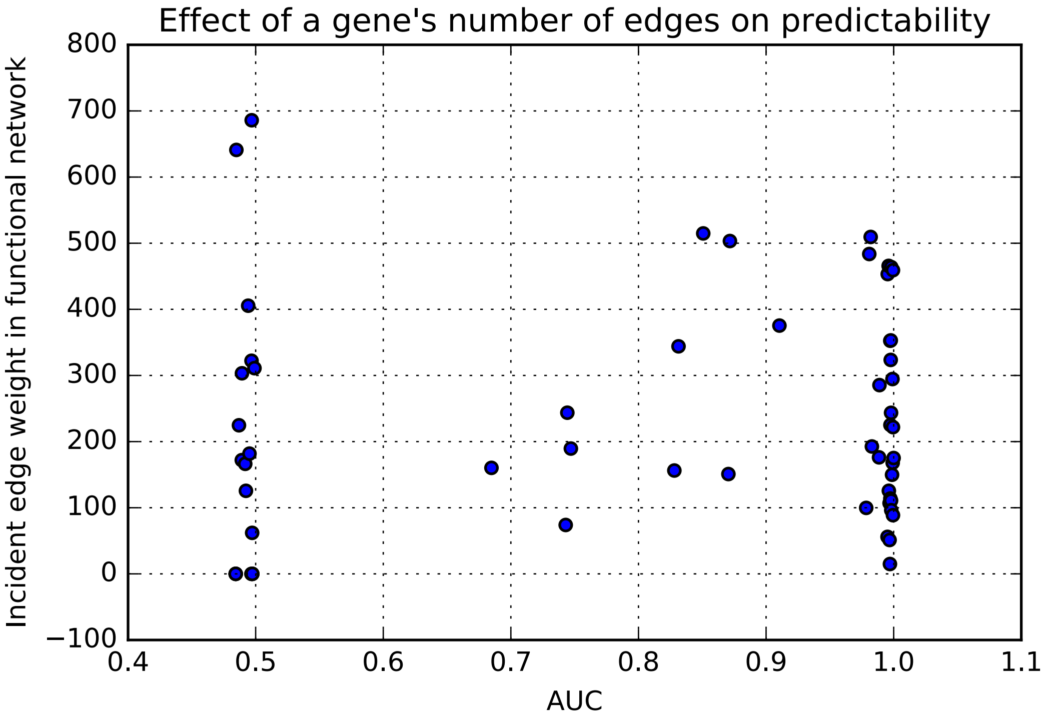

Figure S1. Predictive power as a function of each gene's incident edge weights. A plot of the total incident edge weight of each seed gene against the corresponding predictive power as measured by AUC shows that random AUC values do not correspond with genes that simply have no edges above background in the functional network. Shown here is the case for phenotypic enhancement in human.
