# Supplementary material for: Predictability of Genetic Interactions from Functional Gene Modules
Source: G3 (Bethesda). 2016 Dec 21;7(2):617–24. doi: 10.1534/g3.116.035915 (PMC5295606; doi:10.1534/g3.116.035915)
Supplement: Supplementary file 2 [file 617FigureS2.pdf]

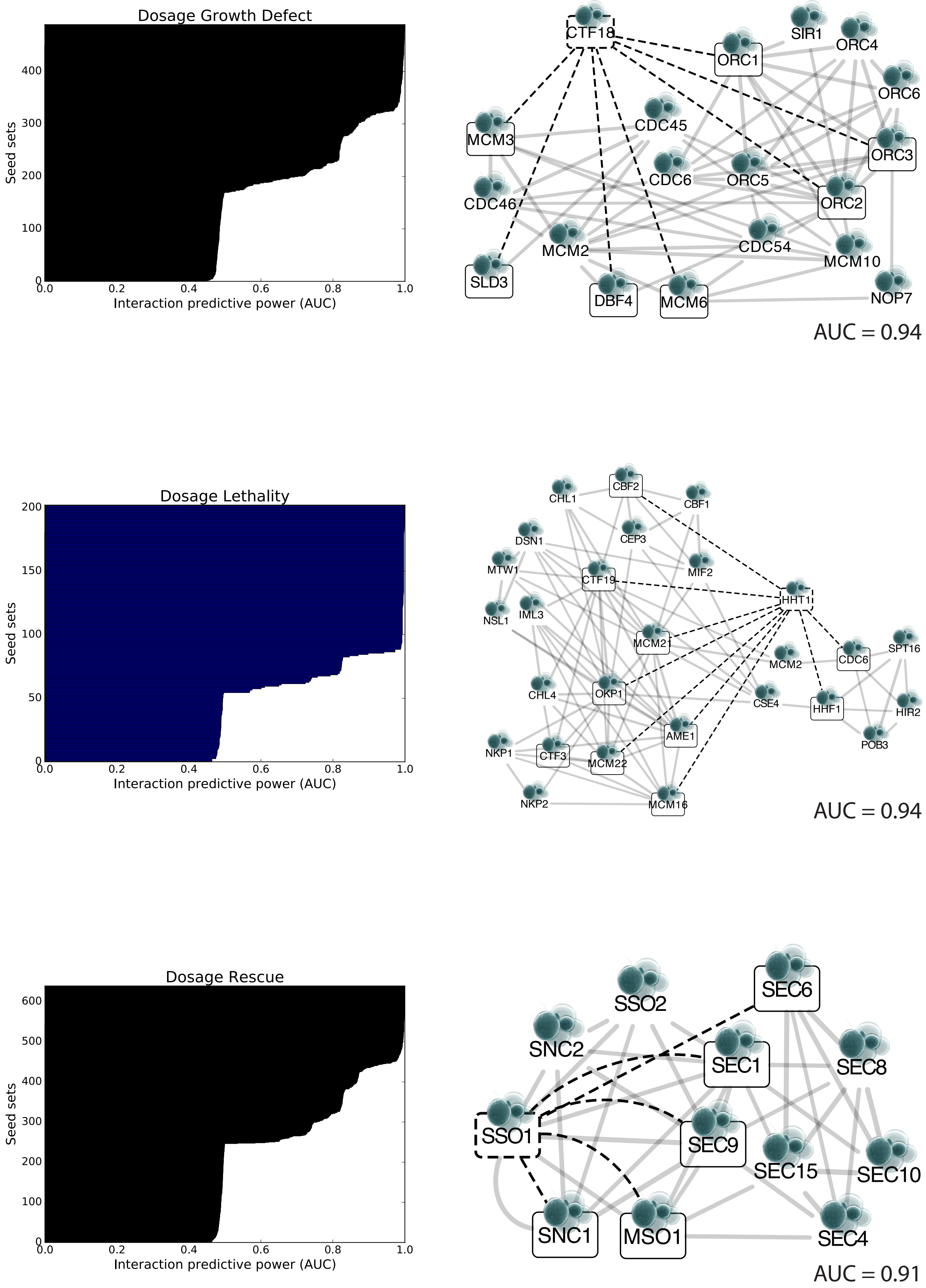
Dosage Growth Defect
Seed sets
Interaction predictive power (AUC)
0.0
0.2
0.4
0.6
0.8
1.0
0
100
200
300
400
CTF18
SIR1
ORC4
ORC1
ORC6
MCM3
CDC45
ORC3
CDC6
ORC5
CDC46
ORC2
MCM2
CDC54
MCM10
SLD3
DBF4
MCM6
NOP7
AUC = 0.94
Dosage Lethality
Seed sets
Interaction predictive power (AUC)
0.0
0.2
0.4
0.6
0.8
1.0
0
50
100
150
200
CBF2
CHL1
CBF1
DSN1
CEP3
MTW1
MIF2
CTF19
HHT1
NSL1
IML3
MCM21
CDC6
SPT16
MCM2
CHL4
OKP1
CSE4
HHF1
HIR2
AME1
POB3
NKP1
CTF3
MCM22
NKP2
MCM16
AUC = 0.94
Dosage Rescue
Seed sets
Interaction predictive power (AUC)
0.0
0.2
0.4
0.6
0.8
1.0
0
100
200
300
400
500
600
SEC6
SSO2
SNC2
SEC1
SEC8
SSO1
SEC9
SEC15
SEC10
SNC1
MSO1
SEC4
AUC = 0.91

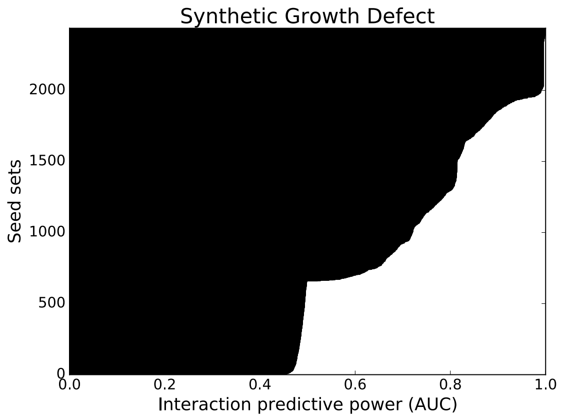
Synthetic Growth Defect
Seed sets
0
500
1000
1500
2000
0.0
0.2
0.4
0.6
0.8
1.0
Interaction predictive power (AUC)

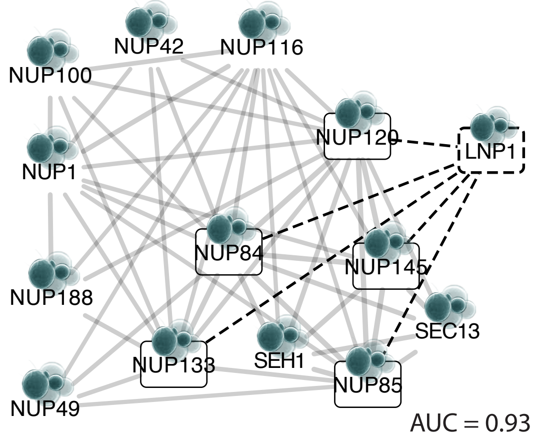
NUP42
NUP116
NUP100
NUP120
LNP1
NUP1
NUP84
NUP145
NUP188
SEC13
NUP133
SEH1
NUP85
NUP49
AUC = 0.93

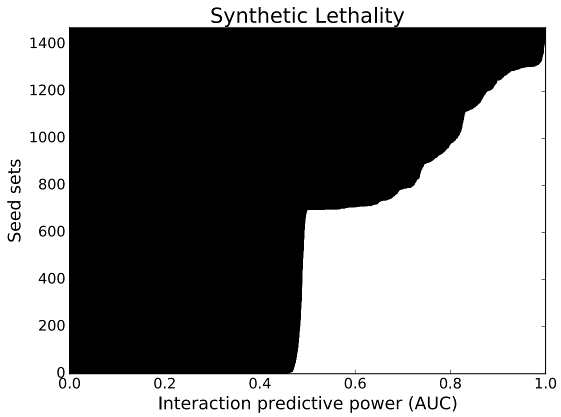
Synthetic Lethality
Seed sets
0
200
400
600
800
1000
1200
1400
0.0
0.2
0.4
0.6
0.8
1.0
Interaction predictive power (AUC)

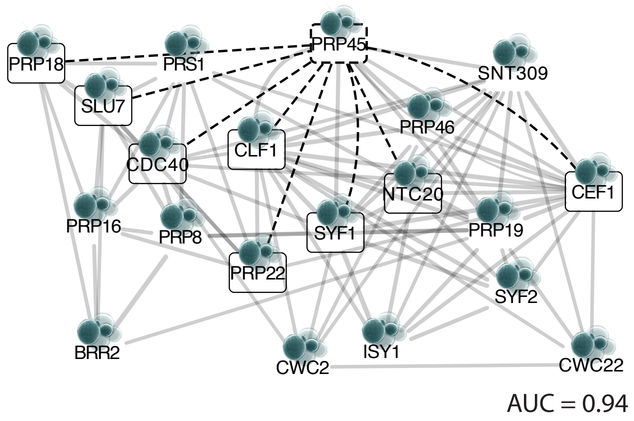
PRP18
PRS1
PRP45
SNT309
SLU7
PRP46
CLF1
CDC40
NTC20
CEF1
PRP16
PRP8
SYF1
PRP19
PRP22
SYF2
BRR2
CWC2
ISY1
CWC22
AUC = 0.94

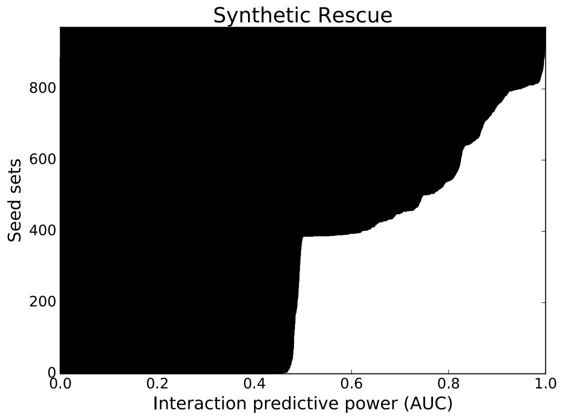
Synthetic Rescue
Seed sets
0
200
400
600
800
0.0
0.2
0.4
0.6
0.8
1.0
Interaction predictive power (AUC)

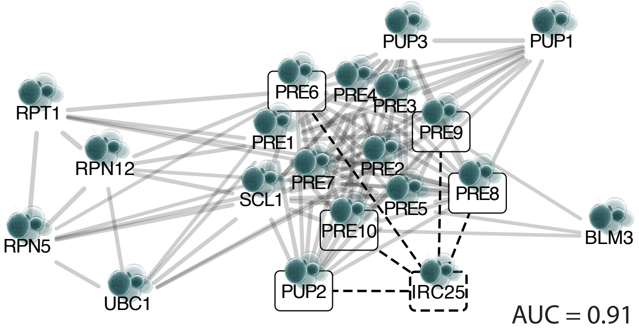
PUP3
PUP1
PRE6
PRE4
PRE3
RPT1
PRE9
PRE1
RPN12
PRE2
PRE7
PRE8
SCL1
PRE5
PRE10
BLM3
RPN5
UBC1
PUP2
IRC25
AUC = 0.91

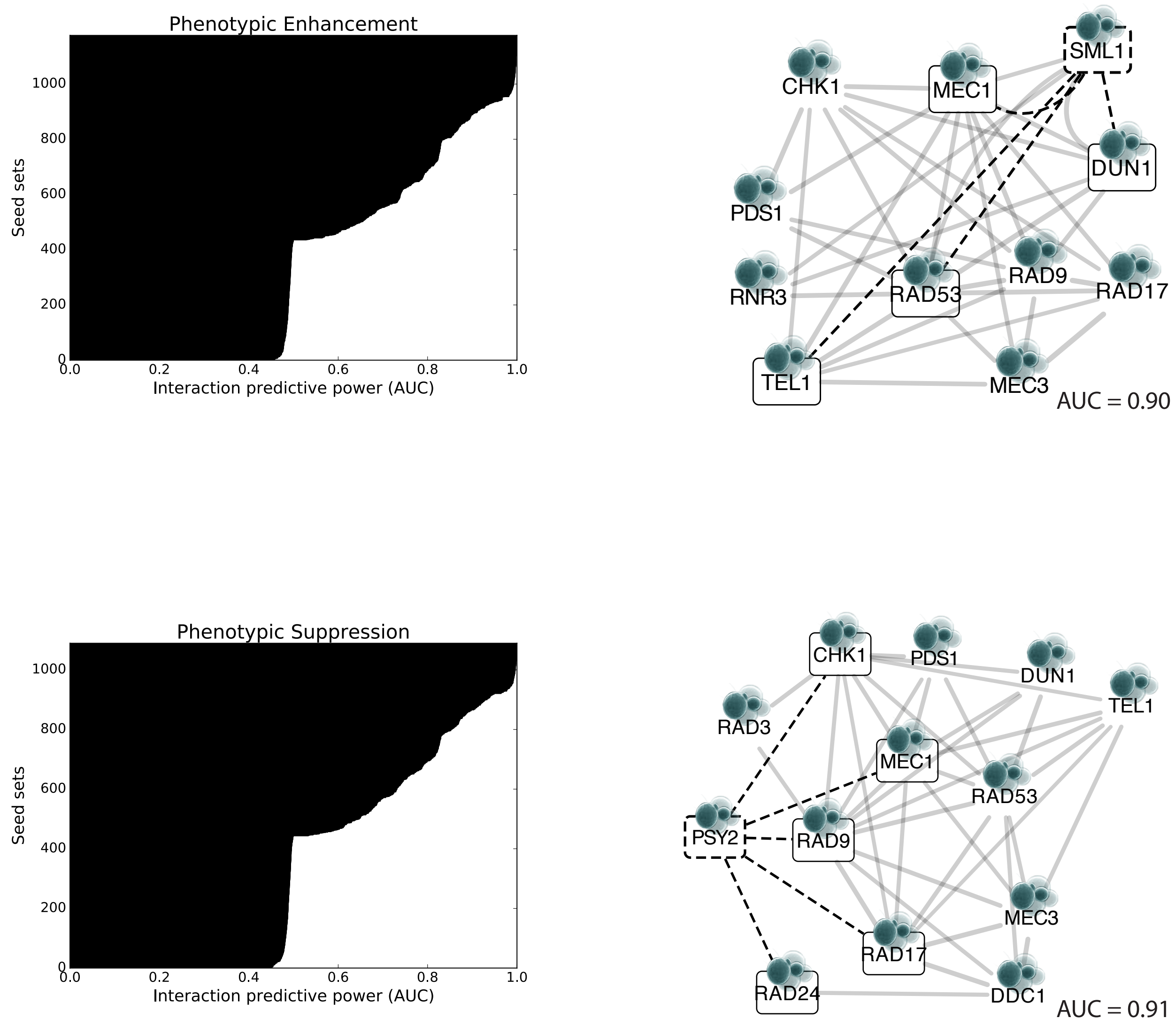

Figure S2. Yeast functional networks are predictive for diverse genetic interactions. The horizontal bar plots on the left hand side show the number of predictive functional net clusters for each genetic interaction. Each bar is a seed set, which is a group of genes all known to share genetic intearctions with a particular gene. If the gene group is clustered in the yeast functional network, then it is predictive of additional novel genetic interactions as measured by the AUC, the area under a receiver operating characteristic curve. On the right are highly predictive functional net clusters for each genetic interaction type, with genetic interactions and functional connections indicated by dotted and solid lines, respectively. For clarity, only edges above a weight cutoff and genes with more than one interaction to the seed set are shown. In the case of phenotypic suppression for instance, the seed set consists of the gene group CHK1, MEC1, RAD9, RAD17 and RAD24, which are all known to phenotypically suppress PSY2.
