# Supplementary material for: Predictability of Genetic Interactions from Functional Gene Modules
Source: G3 (Bethesda). 2016 Dec 21;7(2):617–24. doi: 10.1534/g3.116.035915 (PMC5295606; doi:10.1534/g3.116.035915)
Supplement: Supplementary file 3 [file 617FigureS3.pdf]

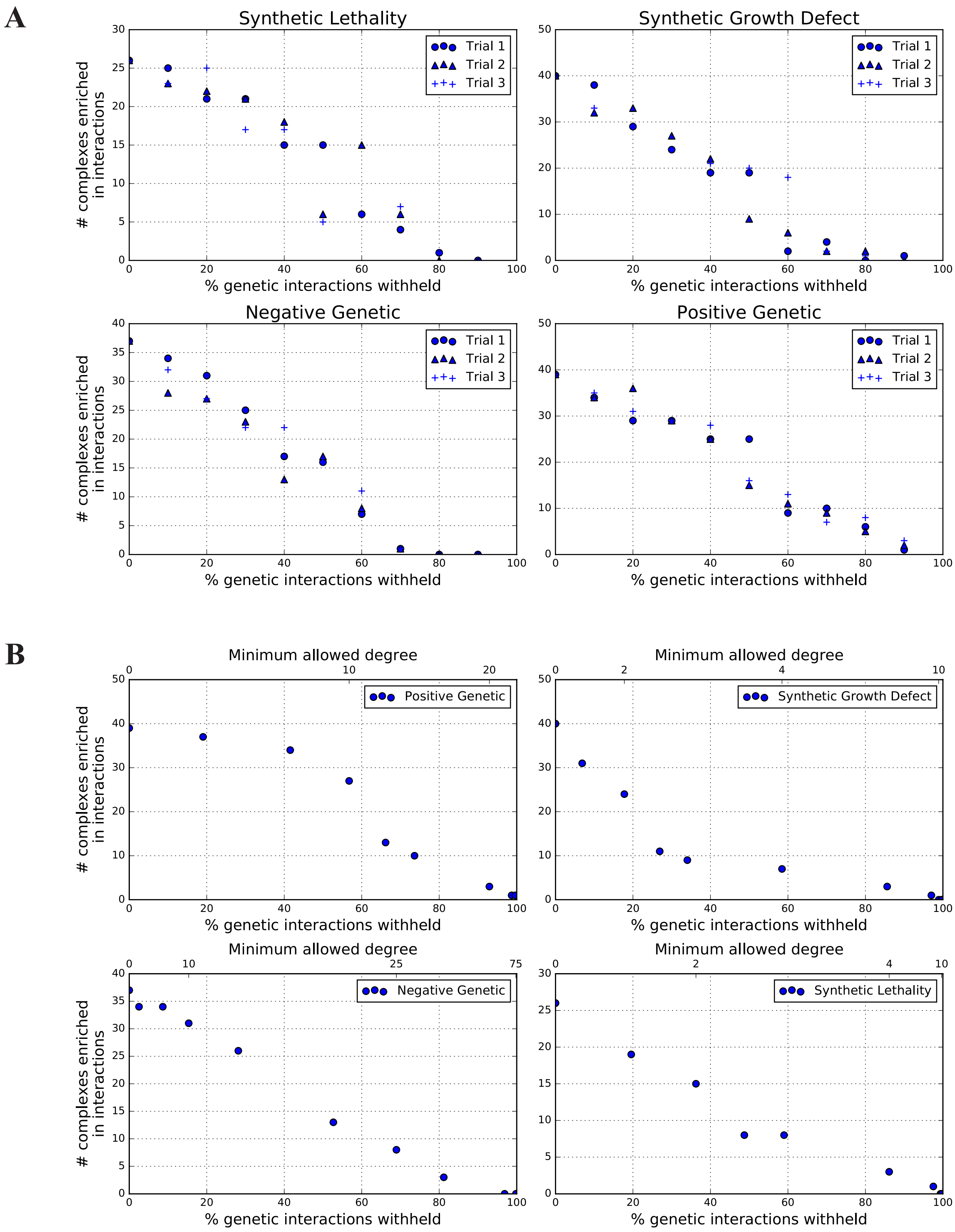

Figure S3. Alternative subsampling methods find predictability even when known genetic interactions are sparse. (A) In edge-based subsampling, synthetic growth defect and lethality, and negative and positive genetic interacting gene pairs were withheld uniformly at random over three trials. (B) Considering the same genetic interactions as in (A), except that at each point, genes under a set interaction degree cutoff are removed.
