# Supplementary material for: Predictability of Genetic Interactions from Functional Gene Modules
Source: G3 (Bethesda). 2016 Dec 21;7(2):617–24. doi: 10.1534/g3.116.035915 (PMC5295606; doi:10.1534/g3.116.035915)
Supplement: Supplementary file 4 [file 617TableS1.pdf]

Dosage Growth Defect	Overexpression of one gene in the presence of a mutant causes significant growth defect compared to mutant alone.
Dosage Lethality	Overexpression of one gene in the presence of a mutant is lethal while the mutated gene alone is not.
Dosage Rescue	Growth defect or lethality of a single mutant is rescue by the over-expression of a second gene.
Phenotypic Enhancement	Double mutant shows marked increase in non-growth phenotypic effect in comparison to either single mutant.
Phenotypic Suppression	Double mutant shows marked decrease in non-growth phenotypic effect in comparison to either single mutant.
Synthetic Growth Defect	Double mutant shows marked decrease in growth in comparison to either single mutant.
Synthetic Rescue	Growth defect or lethality of a single mutant is rescued by the mutation in a second gene.
Synthetic Lethality	Double mutant is lethal while either single mutant is non-lethal.
Negative Genetic	Double mutant shows marked decrease in growth in comparison to either single mutant, as determined by high-throughput assays with growth scores.
Positive Genetic	Double mutant shows marked increase in growth in comparison to either single mutant, as determined by high-throughput assays with growth scores.

Table 1: **Table S1. Definitions of types of genetic interaction.** The types of genetic interactions described above are defined by BioGRID, `http://thebiogrid.org`.
